# Antimelanogenesis Effects of Theasinensin A

**DOI:** 10.3390/ijms22147453

**Published:** 2021-07-12

**Authors:** Hye Yeon Lim, Eunji Kim, Sang Hee Park, Kyung Hwan Hwang, Donghyun Kim, You-Jung Jung, Spandana Rajendra Kopalli, Yong Deog Hong, Gi-Ho Sung, Jae Youl Cho

**Affiliations:** 1 Department of Biocosmetics, Sungkyunkwan University, Suwon 16419, Korea; gosl177@naver.com (H.Y.L.); 84701@naver.com (S.H.P.); 2 Department of Integrative Biotechnology and Biomedical Institute for Convergence at SKKU (BICS), Sungkyunkwan University, Suwon 16419, Korea; im144069@gmail.com; 3 Basic Research & Innovation Division, R&D Center, AmorePacific Corporation, Yongin 17074, Korea; khhwang@amorepacific.com (K.H.H.); dhkim417@amorepacific.com (D.K.); hydhong@amorepacific.com (Y.D.H.); 4 Biological Resources Utilization Department, National Institute of Biological Resources, Incheon 22689, Korea; yjjung0@korea.kr; 5 Department of Integrative Bioscience and Biotechnology, Sejong University, Seoul 05006, Korea; spandanakopalli@gmail.com; 6 Department of Microbiology, Biomedical Institute of Mycological Resource, International St. Mary’s Hospital and College of Medicine, Catholic Kwandong University, Simgokro, 100 Gil, 7, Seo-gu, Incheon 22711, Korea

**Keywords:** theasinensin A, melanogenesis, MC1R, cAMP, CREB

## Abstract

Theasinensin A (TSA) is a major group of catechin dimers mainly found in oolong tea and black tea. This compound is also manufactured with epigallocatechin gallate (EGCG) as a substrate and is refined after the enzyme reaction. In previous studies, TSA has been reported to be effective against inflammation. However, the effect of these substances on skin melanin formation remains unknown. In this study, we unraveled the role of TSA in melanogenesis using mouse melanoma B16F10 cells and normal human epidermal melanocytes (NHEMs) through reverse transcription polymerase chain reaction (RT-PCR), Western blotting analysis, luciferase reporter assay, and enzyme-linked immunosorbent assay analysis. TSA inhibited melanin formation and secretion in α-melanocyte stimulating hormone (α-MSH)-induced B16F10 cells and NHEMs. TSA down-regulated the mRNA expression of *tyrosinase* (*Tyr*), *tyrosinase-related protein 1* (*Tyrp1*), and *Tyrp2*, which are all related to melanin formation in these cells. TSA was able to suppress the activities of certain proteins in the melanocortin 1 receptor (MC1R) signaling pathway associated with melanin synthesis in B16F10 cells: cyclic adenosine monophosphate (cAMP) response element-binding protein (CREB), protein kinase A (PKA), tyrosinase, and microphthalmia-associated transcription factor (MITF). We also confirmed α-MSH-mediated CREB activities through a luciferase reporter assay, and that the quantities of cAMP were reduced by TSA in the enzyme linked immunosorbent assay (ELISA) results. Based on these findings, TSA should be considered an effective inhibitor of hyperpigmentation.

## 1. Introduction

The skin is the largest tissue consisting of multiple epithelial tissues that protect muscles and organs, according to the definition of animal anatomy and dermatology [1]. The skin is a very important organization that protects the body from pathogens and maintains constancy [2] of insulation, body temperature control, sensory functions, the synthesis of vitamins, and the protection of vitamin B folate [3]. The skin is composed of the epidermis, dermis, and subcutaneous layers. Among them, the epidermis is the outermost layer that consists of exfoliating cells, Langerhans cells, and melanin-forming cells [4,5]. Between the epidermis and dermis, melanocytes are evenly distributed in the layers of keratinocytes [6]. When the skin is exposed to UV light, keratinocytes produce α-melanocyte stimulating hormone (α-MSH), which induces the melanocortin 1 receptor (MC1R) signaling pathway in melanocytes [7]. The activation of the MC1R signaling pathway by α-MSH leads to the activation of the cyclic adenosine monophosphate (cAMP) signaling pathway, which is mainly associated with pigment production [8,9]. In sequence, activated microphthalmia-associated transcription factor (MITF) transcribes melanin synthesis-related genes: Mitf itself, Tyrp1, Tyrp2, and Tyr [10,11,12]. Moreover, MITF is essential for melanin formation, the development of melanin-producing cells, and long-term cell survival [13]. Tyrosine is a substrate that is converted into L-Dopaquinone by tyrosinase. L-Dopaquinone then produces dark-brown and red melanin by automatic oxidation and enzyme reactions [11,14]. Formed pigment or melanin protects the skin from UV irradiation by absorbing some of the irradiation [15,16]. Melanin-forming cells also contain DNA-repairing enzymes that restore UV damage; skin cancer is more likely to develop if there is a deficiency in these enzymes [17,18]. Direct exposure to UV light could be a health risk. Acute exposure to UV light causes sunburn or local immunosuppression; however, these are reversible [19,20]. The effects of chronic UV irradiation include vascular damage, atrophy, and fibrosis, and can cause various diseases such as skin cancer, skin lesions, and actinic keratosis [20,21,22,23]. A melanoma is a malignant tumor of melanocytes that produces melanin pigments. It can occur anywhere in the region where melanocytes exist, most often on the skin, and continuously produces melanin [24]. 

**Figure 1:**
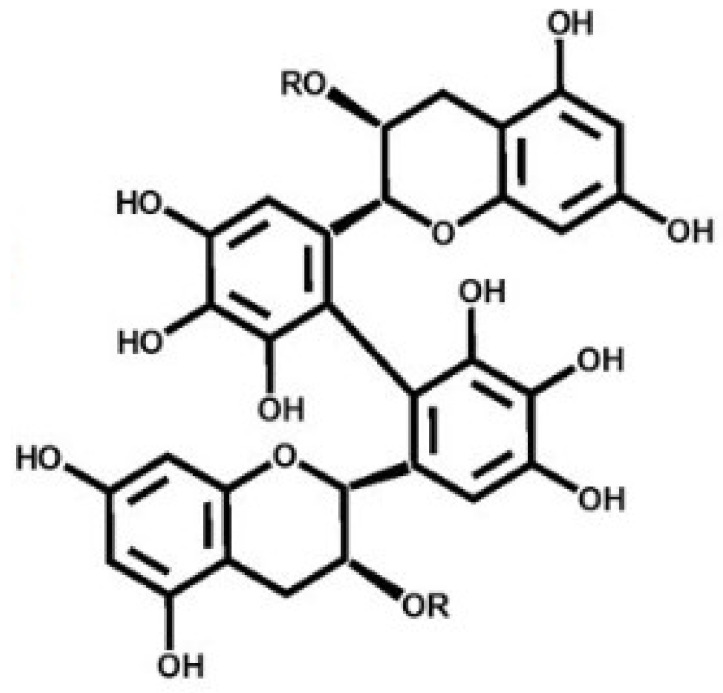
Structure of theasinensin A (TSA). TSA was synthesized by the enzymatic reaction of epigallocatechin gallate (EGCG).

Theasinensin A (TSA) (Figure 1) is a polyphenol flavonoid created during fermentation through the oxidation of epigallocatechin gallate and is a bioactive compound in oolong tea [25,26,27]. In previous studies, mitogen-activated protein kinase kinase (MAPKK) and extracellular signal-regulated kinase (ERK) have been suggested as potential targets for TSA to inhibit inflammatory signaling [28,29], but there have been no reports on the effects of TSA on the creation of melanin on the skin. Therefore, the purpose of this study was to demonstrate the mechanisms of TSA in the inhibition of melanin formation.

In this study, we examined the effects of TSA on melanogenesis using B16F10 murine melanoma cells. Our research revealed that TSA regulates the cyclic adenosine monophosphate (cAMP) element binding (CREB) signaling pathway.

## 2. Results

### 2.1. Effect of Theasinensin A on Melanin Produced by B16F10 Melanoma Cells and Human Epidermal Melanocytes

**Figure 2:**
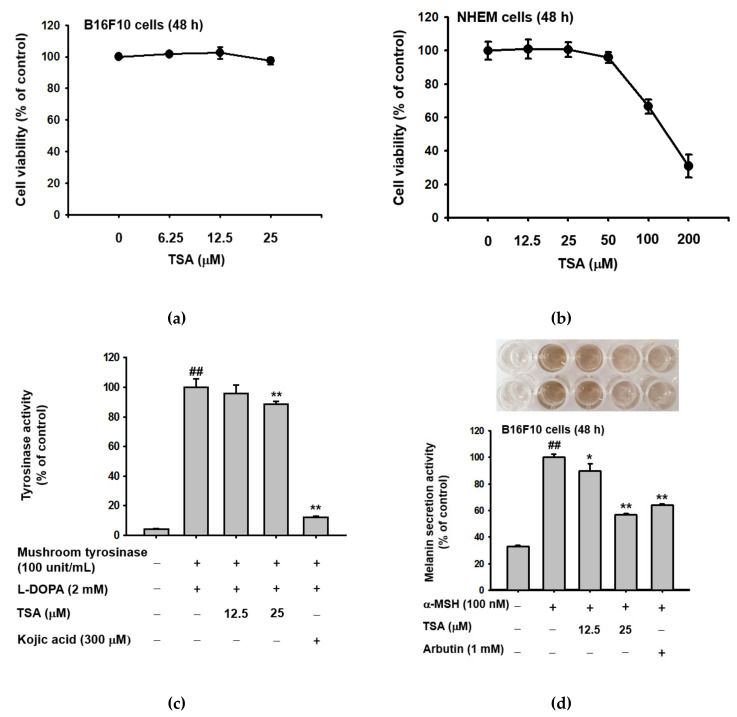

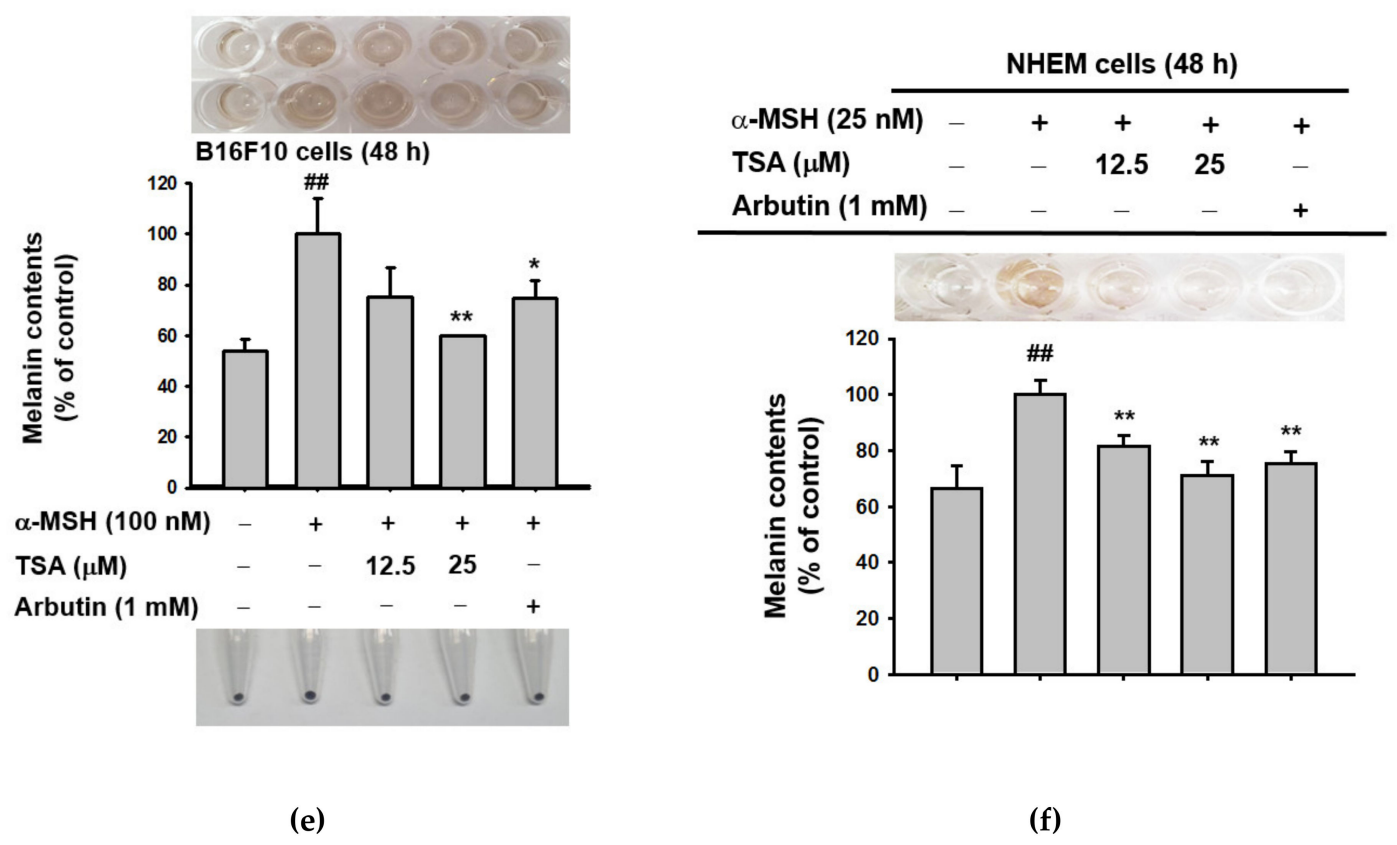
The antimelanogenesis effect of TSA in murine melanoma cells (B16F10). (**a**,**b**) B16F10 cells and NHEMs were treated with TSA (6.25–200 µM) for 48 h, and cell viability was analyzed using MTT assay (*n* = 5 from 3 independent experiments). (**c**) Mushroom tyrosinase was reacted with TSA (6.25–25 µM) or kojic acid (300 μM), and L-DOPA was added. Tyrosinase activity was analyzed by measuring absorbance at 475 nm (*n* = 4 from 3 replicates). (**d**–**f**) B16F10 cells and MHEMs were treated with TSA (12.5–25 µM) or arbutin (1 mM) for 48 h. Cell-cultured media were collected for melanin secretions by measuring absorbance at 470 nm (*n* = 4 from 3 independent experiments). The intracellular melanin contents were determined by measuring optical density at 450 nm with 3 replications. +: indicates treatment, −: indicates non-treatment. For all applicable experiments, statistical significance was evaluated using the Mann–Whitney U test. ## *p* < 0.01 compared with the normal group, * *p* < 0.05 compared with the control group, ** *p* < 0.01 compared with the control group.

We explored the role of TSA in melanin production in both B16F10 melanoma cells and normal human epidermal melanocytes (NHEMs). Firstly, we assessed the cytotoxic dose of TSA with an MTT assay and determined that TSA did not exhibit cell toxicity in the concentration range of 6.25–25 µM for B16F10 cells, or 12.5–50 M for NHEMs for 48 h (Figure 2a,b). Tyrosinase is an important molecule in the melanogenesis process and consists of a copper-containing enzyme that is present in plant and animal tissues and catalyzes the production of melanin and other pigments from tyrosine by oxidation [30,31]. Therefore, we investigated the tyrosinase inhibitory activity of TSA using mushroom tyrosinase and L-3,4-dihydroxyphenylalanine (L-DOPA); mushroom tyrosinase activity was inhibited by TSA. This result indicates that TSA indeed affects tyrosinase activity (Figure 2c). To confirm the effects of TSA on melanin secretion and content in B16F10 cells and NHEMs, we treated TSA or arbutin as a positive control with α-MSH, a melanocyte-stimulate hormone, to induce melanogenesis. The melanin-secreted levels from both B16F10 melanoma cells and NHEMs were significantly inhibited by TSA (12.5–25 µM), and the production of intracellular melanin contents was suppressed in a dose-dependent manner (Figure 2d–f).

### 2.2. Effect of TSA on the Expression of Genes Related to Melanogenesis

**Figure 3:**
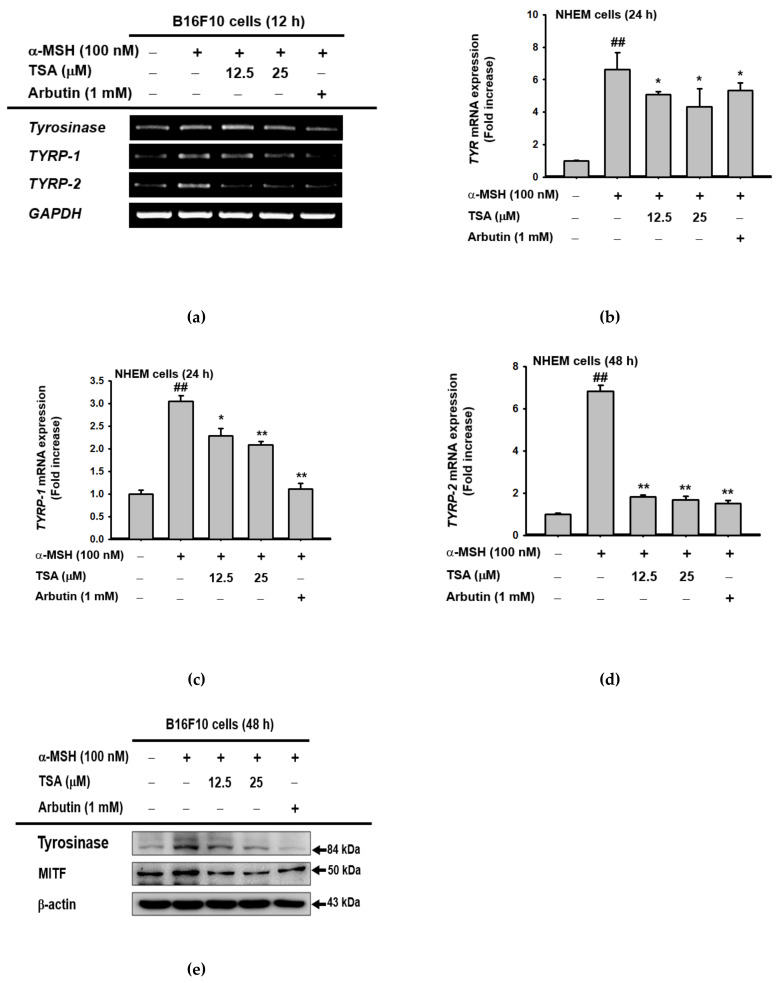
The effect on transcriptional events of melanogenesis. (**a–d**) The mRNA expression of *Tyr*, *Tyrp1*, and *Tyrp2* in B16F10 or NHEMs treated with TSA (12.5–25 µM) or arbutin (1 mM) in response to α-MSH for 24 or 48 h were determined using RT-PCR and real-time PCR analyses. (**e**) Protein levels of tyrosinase and MITF in TSA (12.5–25 µM)- or arbutin (1 mM)-treated B16F10 cells, in response to α-MSH for 48 h of TSA, were determined using Western blotting analysis. For all applicable experiments, statistical significance was evaluated using the Mann–Whitney U test. All experiments were performed at least three times. +: indicates treatment, −: indicates non-treatment. ## *p* < 0.01 compared with the normal group, * *p* < 0.05 and ** *p* < 0.01 compared with the control group.

To investigate which melanogenesis-related genes were regulated by TSA, B16F10 cells and NHEMs were treated with TSA or arbutin with α-MSH in order to induce mRNA expression. The treatment of B16F10 cells and NMEMs with TSA reduced the mRNA expression levels of *Tyr*, *Tyrp1*, and *Tyrp2* (Figure 3a–d). Additionally, increased protein levels of tyrosinase and MITF during α-MSH exposure were also inhibited by TSA (Figure 3e). These outcomes supported previous results that showed a reduction in melanin synthesis via TSA.

### 2.3. Effect of TSA on the cAMP/CREB Signaling Pathway through Regulation of Melanogenesis

**Figure 4:**
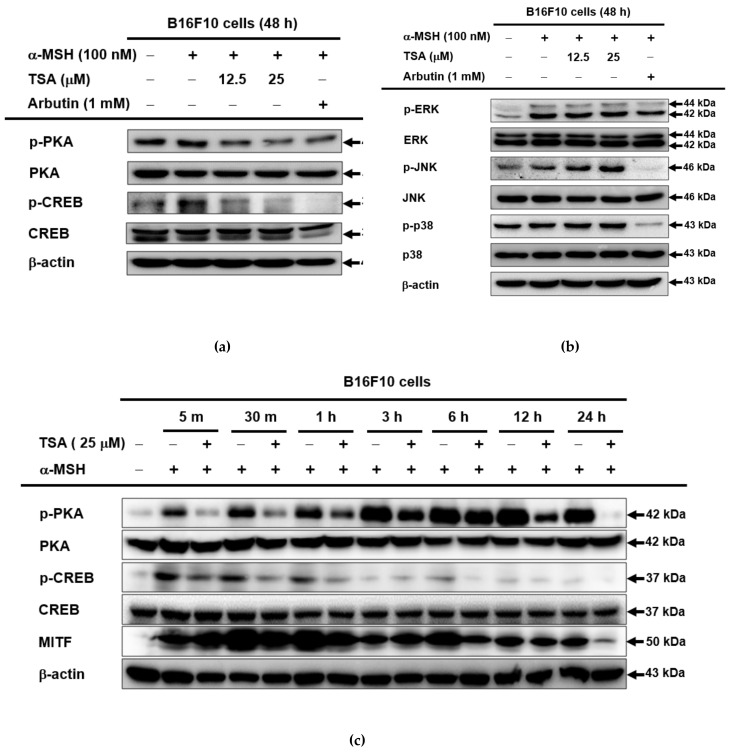

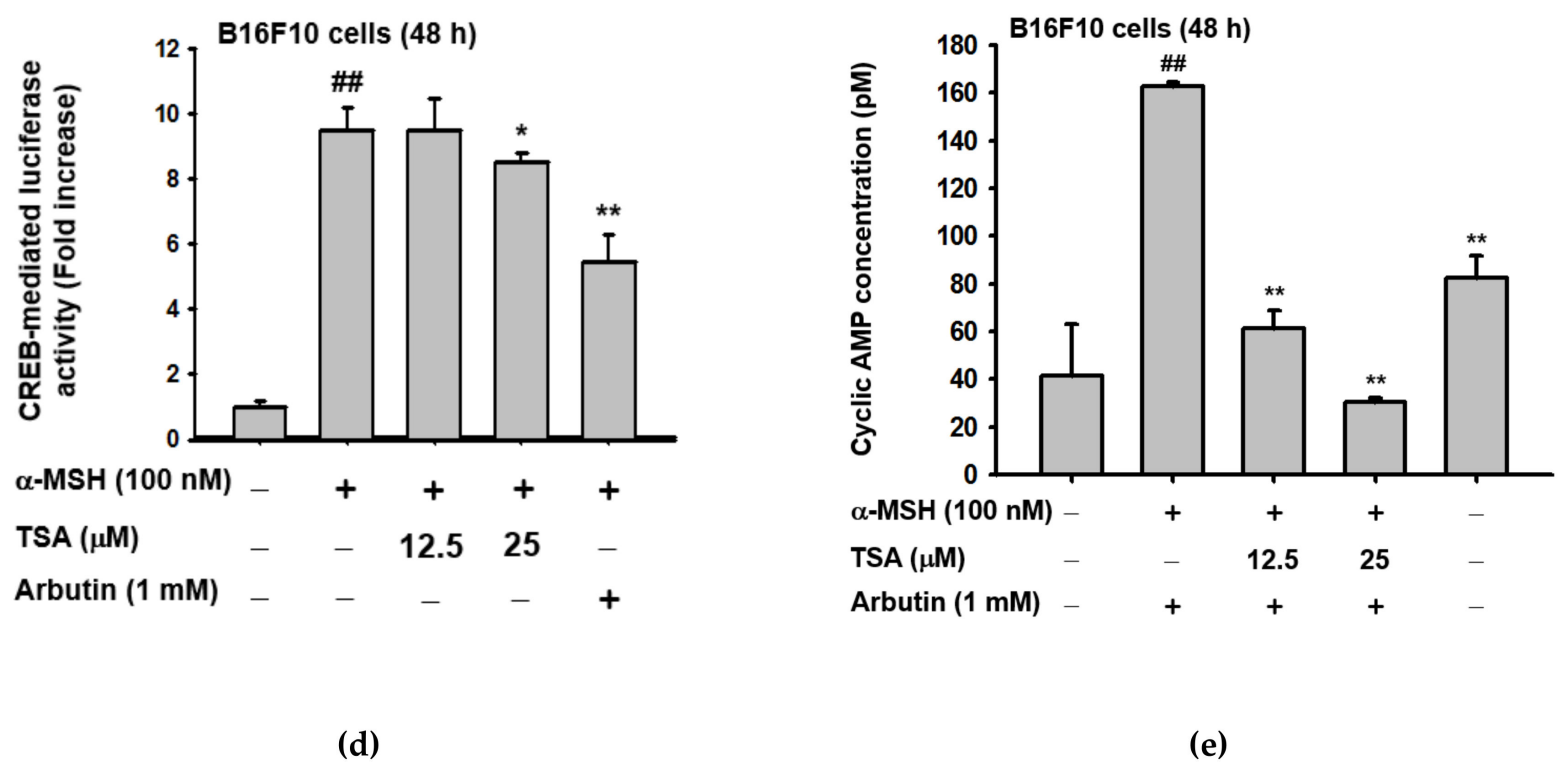
Regulation of the cAMP/CREB signaling pathway of TSA in B16F10 cells. (**a**–**c**) Protein levels of various cyclic adenosine monophosphate (cAMP) signaling proteins and MAPK signaling proteins in B16F10 cells pretreated with TSA (12.5–25 µM) or arbutin (1 mM) in response to α-MSH at indicated time points were determined using Western blotting analysis. (**d**) Activation of CREB in B16F10 cells treated with TSA (12.5–25 µM) or arbutin (1 mM) was determined using the CREB-mediated luciferase system (*n* = 4 with three experimental replicates). (**e**) Levels of cAMP in B16F10 cells treated with TSA (12.5–25 µM) or arbutin (1 mM) were measured using an ELISA assay (*n* = 4). For all applicable experiments, statistical significance was evaluated using the Mann–Whitney U test. ## *p* < 0.01 compared with the normal group, * *p* < 0.05 and ** *p* < 0.01 compared with the control group.

In order to dissect the molecular mechanism of TSA underlying melanogenesis, we subsequently examined the cAMP signaling pathway by Western blotting analysis. TSA inhibited phosphorylation of CREB and PKA (Figure 4a), but there was no alteration in MAPK signaling (Figure 4b). The suppressive patterns of p-PKA and p-CREB levels were also seen from earlier time points (from 5 min to 24 h), as shown in Figure 4c. Synthetically, it is suggested that TSA exhibits antimelanogenesis effects through the regulation of the cAMP signaling pathway, but not the AP-1 signaling pathway. 

We further confirmed the antimelanogenic effect of TSA by regulating the cAMP/CREB signaling pathway in B16F10 melanoma cells. To measure CREB transcriptional activity, we conducted a CREB-mediated luciferase assay in B16F10 cells, as reported previously [32]. TSA at 25 µM significantly decreased CREB-mediated luciferase activity in B16F10 cells (Figure 4d). Moreover, we measured the expression level of cAMP in cells by ELISA analysis because CREB activation relies on cAMP synthesis [33]. It was confirmed that TSA decreased the concentration of cAMP in B16F10 cells in a dose-dependent manner (Figure 4e).

## 3. Discussion

**Figure 5:**
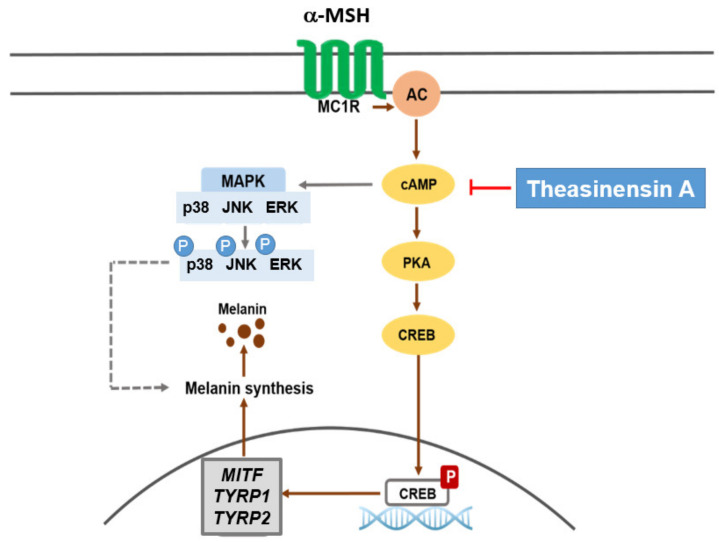
Mechanism of melanogenesis regulated by TSA. Treatment with TSA suppressed melanin Figure 1. and *Tyrp2* through the downregulation of the cAMP pathway in α-MSH-treated B16F10 cells and NHEMs. α-MSH: α-melanocyte stimulating hormone, MC1R: melanocortin 1 receptor, AC: adenylyl cyclase, cAMP: cyclic adenosine monophosphate, PKA: protein kinase A, CREB: cAMP response element-binding protein, MAPK: mitogen-activated protein kinase, JNK: c-Jun-N-terminal kinase, ERK: extracellular signal-regulated kinase, *MITF*: microphthalmia-associated transcription factor, *TRRP1*: *tyrosinase-related protein 1*, and *TYRP2*: *tyrosinase-related protein 1*. Arrows indicate positive signal. Lines with theasinensin A indicate inhibitory signal.

In this study, we determined the antimelanogenesis effect of TSA in B16F10 murine melanoma cells and NHEMs stimulated by α-MSH at non-toxic concentrations (0, 6.25, and 12.5 μM). We stimulated B16F10 cells and NHEMs with α-MSH, which activated various transcription factors and enzymes to induce melanin production [34]. TSA inhibited melanogenesis through the cAMP/CREB signaling pathway (Figure 2d–f and Figure 4d,e). TSA downgraded the mRNA of Tyr, Tyrp1, and Tyrp2, which are important genes in the process of melanin production in DOPA chrome (Figure 3a–d). The antimelanogenesis activities of TSA in B16F10 murine melanoma cells and NHEMs are summarized in Figure 5.

Melanogenesis is a process involving the catalysis of tyrosine by tyrosinase, TYRP1, TYRP2/dopachrome tautomerase (DCT), and MITF. Tyrosine is involved in the formation of melanin as a substrate of tyrosine. Phosphoric acidification of MITF increases the tyrosinase enzyme, an enzyme that oxidizes tyrosine into DOPA, through DOPA as a substrate, and into DOPA quinone [35]. These results indicate that TSA regulates melanin secretion and intracellular melanin content in B16F10 cells by inhibiting the cAMP pathway. Tyrosinases are mediated in this process, and it is thought that the suppression of tyrosinase activity is an effective strategy for the suppression of melanin formation [1,36].

MAPKs consisting of ERK, JNK, and p38 are important adjuncts to melanin formation. MAPKs induce the phosphoric acidification of MITF [37], and MITF expression is activated through c-Kit/MAPK signaling [38]. The expression of MITF and tyrosinase is triggered by p38/MAPK and is related to CREB activity [39,40]. In contrast, the ERK protein induces the degradation of MITF, while p38 blocks melanogenesis by degrading tyrosinase [41,42,43,44]. MAPK proteins exhibit conflicting effects on melanogenesis, but are considered as anti-melanogenic targets in several studies. Several anti-melanogenic substances from natural products such as beauvericin, maclurin, and dihydromyricetin have inhibited both PKA and MAPK activation [40,45,46]. However, in this study, TSA reduced PKA/CREB signaling and not MAPK signaling, implying a different mode of action due to a different chemical structure. The ethanolic extract of *Melia azedarach* L. solely affects the cAMP/PKA/CREB pathway in melanogenesis, while resorcinol from argan oil has an anti-melanogenic effect by suppressing cAMP signaling instead of MAPKs [41,47], implying that structurally similar compounds could be included in these extracts. Hence, natural substances seem to have diverse regulatory mechanisms that affect melanogenesis based on their structural features. 

The MC1R signaling pathway is an important signaling route for melanin formation [48]. Downstream α-MSH-induced MC1R activation leads to the stimulation of adenylyl cyclase (AC) and the production of cAMP, which activates PKA and intracellular CREB proteins [49]. CREB was activated through phosphorylation and induced the expression of MITF [50]. In addition, MITF was modulated by the phosphoinositide 3-kinase/protein kinase B (PI3K/PKB (AKT)) pathway in α-MSH-stimulated MC1R signaling. The activation of PKB suppresses melanogenesis by downregulating MITF transcription [33,51]. Several compounds were investigated for the role of melanogenesis in the formation of the PI3K/PKB pathway. Hydroxyectonie modulated melanogenesis by the activation of PKB and p38 [52]. The anti-melanogenic effect of a caffeamide derivate was elucidated, and phosphorylated PKB was increased [53]. In this study, the activation of PKB was not examined. However, since cAMP/PKA inhibited PI3K/PKB activity to induce melanogenesis [53,54], there is a possibility that TSA regulates PKB activity based on our results (Figure 4a,c–e). An investigation of the regulatory mechanisms of TSA on α-MSH-stimulated MC1R/PKB signaling would be meaningful for further study. 

UV light can cause various skin diseases and abnormalities such as skin cancer, rashes, wrinkling, and skin pigmentation [55]. Melanin induction under UV irradiation is a type of protective mechanism for the skin to avoid burning, irritations as well as the generation of skin cancer [56]. From this view, anti-pigmentation strategies as cosmetics may increase the risk of skin cancer. Therefore, it seems to be very important to check whether an anti-melanogenic compound increases skin cancer risk or not. Since we are also hoping to develop an anti-pigmentation cosmeceutical with TSA, we tested its potential risk for skin cancer. For this, the protective activity of TSA on UV-induced DNA damage and mutation, a major cause of skin cancer [57], was finally examined. To do this, we employed a bacterial reverse mutation test (Ames test), as reported previously [58]. As Figure S1 shows, TSA reduced the colony formation of *Salmonella typhimurium* upregulated by UVB irradiation at 25 μM, implying that TSA can block UVB-induced DNA mutation. This result strongly suggests that TSA can block DNA damage during UV exposure, which can be beneficial, leading to the reduction of skin cancer risk.

## 4. Materials and Methods

### 4.1. Materials

B16F10 cells were purchased from American Type Culture Collection (Rockville, MD, USA). Normal human epidermal melanocytes (NHEMs) and melanocyte growth medium m2 (c24305) were purchased from the Promocell (Heidelberg, Germany). We purchased 3-(4–5-Dimethylthiazol-2-yl)-2,5-diphenyltetrazolium bromide (MTT) from Amresco (Brisbane, Australia). Theasinensin A (TSA) was supplied from Amorepacific Co. (Seoul, Korea). Dulbecco’s Modified Eagle’s medium (DMEM), fetal bovine serum (FBS), phosphate-buffered saline (PBS), streptomycin, penicillin, and L-glutamine were purchased from Gibco (Grand Island, NY, USA). Sodium dodecyl sulfate (SDS), L-3,4-dihydroxyphenylalanine (L-DOPA), 5-hydroxy-2-hydroxymethyl-4H-pyranone (kojic acid), 4-hydroxyphenyl-β-D-glucopyranoside (arbutin), and a-melanocyte stimulating hormone (α-MSH) were obtained from Sigma Chemical Co. (St. Louis, MO, USA). TRIzol reagent was supplied by Molecular Research Center Inc. (Cincinnati, OH, USA). MuLV reverse transcriptase was purchased from Thermo Fisher Scientific (Waltham, MA, USA). Primers specific for *TYR*, *TYRP1*, *TYRP2*, and *GAPDH* for the reverse transcriptase polymerase chain reaction (RT-PCR) were obtained from Bioneer Inc. (Daejeon, Korea). Specific antibodies for total-forms and phospho-forms of tyrosinase, MITF, CREB, PKA, ERK, JNK, p-p38, and β-actin were purchased from Cell Signaling Technology (Beverly, MA, USA) or Santa Cruz Biotechnology (Santa Cruz, CA, USA). Enhanced chemiluminescence reagents were obtained from Ab Frontier (Seoul, Korea). The luciferase assay system was purchased from Promega (Madison, WI, USA). Cyclic AMP Elisa was purchased from Abcam (Cambridge, UK).

### 4.2. Compound Preparation and Treatment

A stock solution of TSA was prepared by dilution in 25 mM of DMSO. TSA was dissolved in 100% dimethylsulfoxide (DMSO) and then further diluted in the culture medium to prepare the indicated concentrations. Equal amounts of DMSO (0.2 µL DMSO/200 µL medium) were always prepared for the corresponding normal or control groups as a vehicle control.

### 4.3. Cell Culture

B16F10, the murine melanoma cell line, was cultured in DMEM supplemented with 10% FBS and 1% streptomycin (100 mg/mL) and penicillin (100 U/mL) at 37 °C in a 5% CO_2_ humidified incubator. NHEM cells were cultured in M2 media supplemented with 1% penicillin–streptomycin in a 5% CO_2_ incubator at 37 °C.

### 4.4. MTT Assay

In order to determine the non-cytotoxic concentration of TSA in melanocytes, we employed the MTT assay. Since NHME is a normal human fibroblast, a wide range of concentrations of TSA were used to treat the NHMEs. B16F10 cells or NHEM cells (5 × 10^4^ cells/mL) were seeded onto a 96-well plate. Cells were incubated in TSA (6.25–100 µM) for 48 h. A conventional MTT assay was conducted, and absorbance at 570 nm was measured by a Spectra Max 250 microplate reader, as previously described [59]. 

### 4.5. Tyrosinase Activity Assay

TSA (12.5–25 µM) or kojic acid (300 µM) and mushroom tyrosinase (100 unit/mL) were incubated for 30 min in a 5% CO_2_ humidified incubator and treated with L-DOPA (40 µg/mL) for 5 min on a 96-well plate. Tyrosinase activity was measured using the absorbance of mixture at 475 nm by a Spectra Max 250 microplate reader. Tyrosinase activity assay was conducted, as previously described [60].

### 4.6. Melanin Secretion and Content Assay

B16F10 cells (0.5 × 10^5^ cells/well) or NHEM cells (2 × 10^5^ cells/mL) were seeded into a 12-well plate and incubated for 24 h. The culture media were replaced with new, fresh media containing TSA (12.5–25 µM) and arbutin (1 mM). The α-MSH (100 nM) was subsequently used to stimulate the cells for 48 h. Afterwards, the secreted culture medium was measured by absorbance at 475 nm using a Spectra Max 250 microplate reader. Harvested cells were lysed by lysis buffer (50 mM Tris HCL pH 7.5, 20 mM β-glycerophosphate pH 7.5, 120 mM NaCl, and 2% NP-40) and centrifuged at 10,000× *g* for 3 min. Concentrated cell pellets were resuspended in 10% DMSO in 1 N NaOH and incubated in a heating block for 10 min at 60 °C. The melanin contents were measured at an absorbance of 405 nm using a Spectra Max 250 microplate reader. A melanin secretion and contents assay was conducted, as previously described [60].

### 4.7. Reverse Transcription Polymerase Chain Reaction (RT-PCR) and Real-Time PCR

To confirm the expression of mRNA related to melanogenesis, B16F10 cells and NHEMs (4 × 10^5^ cells) were treated with TSA (12.5–25 µM) or arbutin (1 mM) with α-MSH as a positive control to stimulate melanogenesis for 12 h. Total RNA was extracted using a TRIzol reagent. cDNA was synthesized from total RNA (1 µg) using MuLV reverse transcriptase, which was produced according to the instructions of the manufacturer. RT-PCR and real-time PCR analyses were conducted, as previously described, with modification [60,61]. Primer sequences used in this study are listed in Table 1. 

### 4.8. Preparation of Total Cell and Lysates

B16F10 cells were treated with TSA (12.5–25 µM) or arbutin (1 mM) with μ-MSH (100 nM) for 48 h. We prepared the total cell lysates, as previously described [62,63]. Cells (1 × 10^6^ cells/mL) were washed with cold PBS and lysed with buffer (20 mM Tris-HCl, pH 7.4, 2 mM EDTA, 2 mM EGTA, 50 mM glycerol phosphate, 1 mM DTT, 2 µg/mL aprotinin, 2 µg/mL leupeptin, 1 µg/mL pepstatin, 50 µM PMSF, 1 mM benzamide, 2% Triton X-100, 10% glycerol, 0.1 mM sodium vanadate, 1.6 mM pervanadate, and 20 mM NaF). Total cell lysates were clarified by centrifugation at 12,000 rpm for 5 min at 4 °C, and stored at −20 °C until used. 

### 4.9. Western Blotting Analysis

Proteins were quantified and separated using SDS-polyacrylamide gel electrophoresis. SDS gel proteins were transferred to polyvinylidene fluoride membranes. The membranes were blocked using 5% bovine serum albumin (BSA) in 0.1% TBST (Tris-base, NaCl and 0.1% Tween 20) at room temperature for 1 h, followed by the incubation with specific primary antibodies for at least 1 h at room temperature. After primary antibody incubation, the membranes were washed with 0.1% TBST 3 times for 10 min each. The membrane was incubated with HRP-linked secondary antibodies in 3% BSA solution for at least 1 h at room temperature and washed with 0.1% TBST 3 times for 10 min each. Phosphorylated and total forms of tyrosinase, MITF, PKA, CREB, ERK, JNK, p38, and *β*-actin were used. To detect target proteins, we used specific antibodies and protein bands were visualized using enhanced chemiluminescence reagents. Western blotting analysis was conducted, as previously described [60,63].

### 4.10. Luciferase Assay

B16F10 cells (1 × 10^5^ cells/well) were seeded for 24 h into a 12-well plate prior to transfection with plasmids (0.8 µg/mL per well), encoding a luciferase gene under a CREB promoter and β-galacosidase as a control by the lipofectamine method, as previously reported [32]. After 24 h of stabilization, the transfected cells were treated with TSA (12.5–25 µM) or arbutin (1 mM) concomitantly with α-MSH (100 nM) for the next 24 h. Luciferase activity was assessed using the Luciferase Assay System (Promega, Madison, WI, USA), as previously reported [64,65]. 

### 4.11. Enzyme-Linked Immunosorbent Assay (ELISA)

B1F10 cells (4 × 10^5^ cells/well) were seeded into a 6-well plate and incubated for 24 h. The culture media was changed into new fresh media containing TSA (12.5–25 µM) and arbutin (1 mM) and incubated for 24 h. The supernatant of the media was collected from each well, and cyclic AMP was measured with an ELISA assay kit (Abcam, Cambridge, UK), following the manufacturer’s instructions.

### 4.12. Statistical Analysis

All data of the experiment are presented as a mean ± standard deviation (SD) of at least three replicates of each experiment. Experimental and control groups were compared by Mann–Whitney test. A *p* value < 0.05 was considered statistically significant (* *p* < 0.05, ** *p* < 0.01). The SPSS program (SPSS Inc., Chicago, IL, USA) was used for statistical analysis. 

## 5. Conclusions

The results of this study showed that TSA suppresses melanin formation control and improves whitening effects in test premises by inhibiting the cAMP signaling pathway, which is managed by balancing between adenylyl cyclase (AC) and cAMP phosphodiesterases (cAMP PDEs) instead of via MAPK signaling and the tyrosinase inhibitory activity level. These results suggest that TSA can be developed as a cosmeceutical material with anti-melanogenic activity, which can be used for skin improvement or as an anti-skin hyperpigmentation agent to treat dyschromia and lentigo, conditions mostly caused by UV irradiation. The exact mechanism of how TSA can increase the cAMP level will be further studied in terms of the activation of AC or the inhibition of cAMP PDEs. Additionally, since it was reported that some plant-derived compounds with anti-melanogenic activities have the possibility to induce cellular senescence [66,67], whether TSA can also cause this phenomenon will likewise be examined.

## Figures and Tables

**Table 1:** Sequences of primers (mouse) used in RT-PCR.

Name	Direction	Sequence (5′ to 3′)	Anneal Temp. (°C)
Mouse			
*Tyr*	Forward	GTCCACTCACAGGGATAGCAG	56
	Reverse	AGAGTCTCTGTTATGGCCGA	
*Tyrp1*	Forward Reverse	ATGGAACGGGAGGACAAACC TCCTGACCTGGCCATTGAAC	57
*Tyrp2*	Forward Reverse	CAGTTTCCCCGAGTCTGCAT GTCTAAGGCGCCCAAGAACT	58
*Gapdh*	Forward Reverse	ACCACAGTCCATGCCATCAC CCACCACCCTGTTGCTGTAG	58
Human			
*Tyr*	Forward Reverse	AACAAGCGAGTCGGATCTGG GACGACACAGCAAGCTCACA	58
*Tyrp1*	Forward Reverse	GTGCCACTGTTGAGGCTTTG ATGGGGATACTGAGGGCTGT	58
*Tyrp2*	Forward Reverse	CTGGCCCCTATTGGTCACAA TGGCAGATCGATGGCATAGC	58
*Gapdh*	Forward Reverse	TCAAGGCTGAGAACGGGAAG TCGCCCCACTTGATTTTGGA	57

## Data Availability

The data used to support the findings of this study are available from the corresponding authors upon request.

## Supplementary Materials

The following are available online at https://www.mdpi.com/article/10.3390/ijms22147453/s1, Figure S1: Anti-mutagenic activity of TSA.

## Abbreviations

TSA	Theasinensin A
AP-1	Activator protein 1
Tyrp1	Tyrosinase-related protein 1
Tyrp2	Tyrosinase-related protein 2
CREB	cAMP response element binding
PKA	Protein kinase A
MITF	Microphthalmia-associated transcription factor
MC1R	Melanocortin 1 receptor
α-MSH	α-Melanocyte-stimulating hormone
NHEM	Normal human epidermal melanocytes
