# Supplementary material for: Antimelanogenesis Effects of Theasinensin A
Source: Int J Mol Sci. 2021 Jul 12;22(14):7453. doi: 10.3390/ijms22147453 (PMC8305159; doi:10.3390/ijms22147453)
Supplement: Supplementary file 1 [file ijms-22-07453-s001.zip › ijms-1274736-supplementary.pdf]

Supplementary material

Antimelanogenesis Effects of Theasinensin A

Hye Yeon Lim [1,a], Eunji Kim [2,a], Sang Hee Park [1,a], Kyung Hwan Hwang [3], Donghyun Kim [3], You-Jung Jung [4], Yong Deok Hong [3], Gi-Ho Sung [5,*] and Jae Youl Cho [1,2,*]

[1] Department of Biocosmetics, Sungkyunkwan University, Suwon 16419, Republic of Korea; gosl177@naver.com (H.Y.L.); 84701@naver.com (S.H.P.); jaecho@skku.edu (J.Y.C.)

[2] Department of Integrative Biotechnology and Biomedical Institute for Convergence at SKKU (BICS), Sungkyunkwan University, Suwon, 16419, Republic of Korea; im144069@gmail.com (E.K.); jaecho@skku.edu (J.Y.C.)

[3] Basic Research & Innovation vision, R&D Center, AmorePacific Corporation, Yongin 17074, Korea; khhwang@amorepacific.com (H.K); dhkim417@amorepacific.com (D.K); emkim@amorepacific.com (E.M.K)

[4] Biological Resources Utilization Department, National Institute of Biological Resources, Incheon 22689, Republic of Korea; yjjung0@korea.kr

[5] Department of Microbiology, Biomedical Institute of Mycological Resource, International St. Mary's Hospital and College of Medicine, Catholic Kwandong University, Simgokro, 100 Gil, 7, Seo-gu, Incheon 22711, Republic of Korea; Email: sung97330@gmail.com

* Correspondence: sung97330@gmail.com (G.-H.S.) Tel.: +82-32-290-2772, and jaecho@skku.edu (J.Y.C.); Tel.: +82-31-290-7868

[a] These authors equally contributed to this work.

Methods.

Bacterial strain

S. typhimurium test strain TA98 from MOLTOX Inc. (NC, USA) was used for the bacterial reverse mutation assay. The strain was stored as stock cultures in ampoules with nutrient broth (OXOID) supplemented with approximately 8% v/v dimethyl sulfoxide (DMSO) in liquid nitrogen.

Bacterial reverse mutation test

The bacterial reverse mutation test was conducted in accordance with the MFDS guideline (Notification No.2013–121). Histidine auxotroph mutant of *Salmonella typhimurium* strains TA98 was cultured in nutrient broth No.2 medium for 8.5 h in a shaking water bath (37°C, 130 rpm). The turbidity of the cultures was measured with a UV/VIS spectrophotometer (660 nm, V-550, Jasco, Japan). Cultures with a density greater than 1×10^9 cells/mL were used in this test. In the presence of metabolic activation, 100 μL of TSA with different concentrations were mixed with 500 μL of S9 mix and 100 μL of pre-incubated

bacterial suspension before UVB irradiation (2 mJ/cm2). These mixtures were incubated in a shaking water bath at 37°C for 20 min. Then, 2 mL of warmed top agar for *Salmonella typhimurium* were added to the TA98 strain. Finally, these mixtures were poured onto minimal glucose agar plates. In the absence of metabolic activation, 500 μL of 0.1 mol/L sodium phosphate buffer (pH 7.4) instead of S9 mix were added, and the rest of procedure was carried out with the same method as above. After the top agar was solidified, the plates were cultured in an incubator at 37°C for 48 h. Triplicate plates were used per dose in the test.

Results

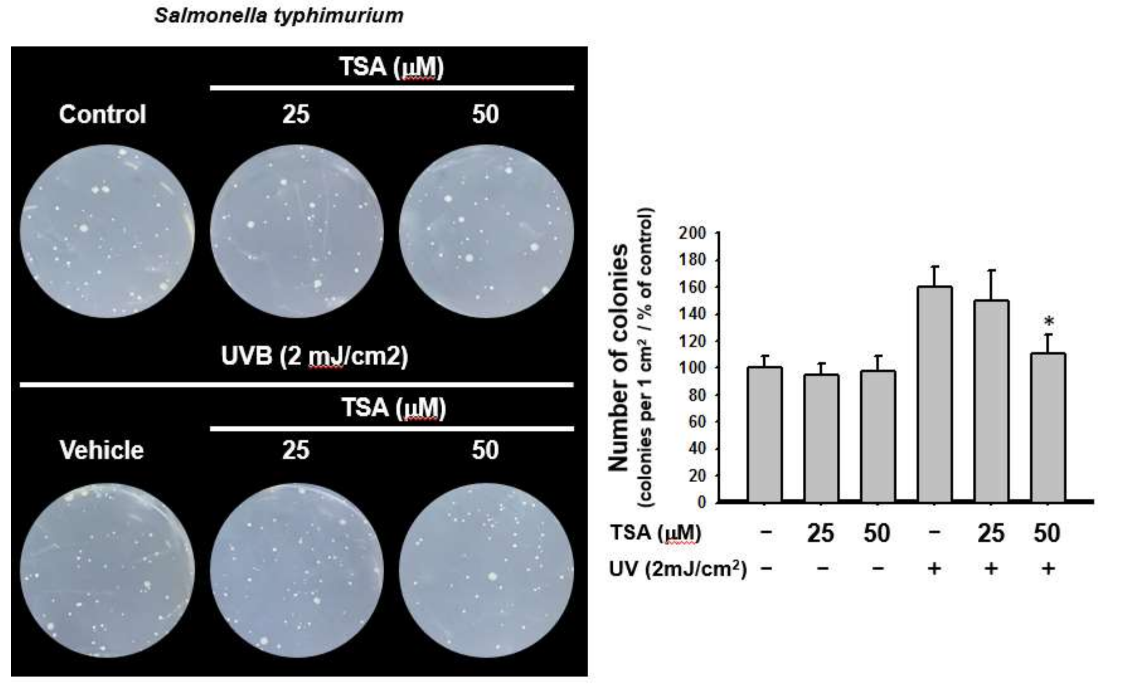

Figure S1. Anti-mutagenic activity of TSA. Anti-mutagenic activity of TSA was observed by Ames test performed with *Salmonella typhimurium* irradiated by UVB (2 mJ/cm^2). Number of colonies grown in agar plate was obtained by counting the colonies on agar plate.
